# Vector Analysis of the Effects of FS-LASIK and Toric ICL for Moderate to High Astigmatism Correction

**DOI:** 10.1155/2018/6952710

**Published:** 2018-07-25

**Authors:** Kaijian Chen, Zongli Hu, Jihan Zhou, Ting Yu, Jie Xu, Ji Bai, Jian Ye

**Affiliations:** Department of Ophthalmology, Research Institute of Surgery and Daping Hospital, Army Medical University, Chongqing, China

## Abstract

**Purpose:**

To estimate the treatment effectiveness of femtosecond-assisted laser in situ keratomileusis (FS-LASIK) and Toric implantable collamer lens (Toric ICL) for moderate and high astigmatism via vector analysis.

**Materials and Methods:**

The study involved 44 eyes from 44 patients who had a preoperative refractive cylinder ≥1.0 diopters (D) and underwent bilateral FS-LASIK or Toric ICL surgery. The examinations included corrected distance visual acuity measurement and subjective refraction before and 3 months after surgery. The astigmatic changes were estimated using vector analysis.

**Results:**

No statistically significant differences were found in cylindrical refraction and percentage of spherical equivalent within 0 D, ±0.50 D, ±1.00 D, and ±1.50 D between the FS-LASIK and Toric ICL groups at 3 months after surgery. The parameters of the vector analysis included intended refractive correction, surgically induced refractive correction, error vector, correction ratio, error ratio, error of magnitude, and error of angle, with no significant differences between the groups. However, error ratio the of the off-axis correction in the FS-LASIK and Toric ICL groups was 4.11 ± 3.02 and 8.11 ± 3.82, respectively, and the difference was significant (*t *= −2.46, *p*=0.02).

**Conclusion:**

Both FS-LASIK and Toric ICL were effective for correcting moderate and high astigmatism, although Toric ICL might produce a larger error of angle than FS-LASIK when an off-axis correction occurs.

## 1. Introduction

Astigmatism is a vital factor leading to visual quality decline besides myopia, and it should be corrected via refractive surgery. Currently, there are two ways of surgery to treat astigmatism combined with myopia, corneal refractive surgery and Toric implantable collamer lens (Toric ICL) surgery [1–3]. Both treatments may be selected for the treatment of moderate and high astigmatism. Generally, corneal refractive surgery requires the ablation of corneal tissue, which could induce a change in corneal biomechanics [4] and lead to high-order aberrations [5]. However, Toric ICL avoids the complications associated with ablation, although the postoperative rotation of the lens could influence visual quality [6, 7]. For corneal refractive surgery, both femtosecond-assisted laser in situ keratomileusis (FS-LASIK) and small incision lenticule extraction (SMILE) have been shown to be safe and effective in the treatment of astigmatism [8]. SMILE has the advantages of fast nerve recovery and biomechanical stability but lacks pupil tracking and iris recognition technology, which can be used to obtain more accurate alignment and correction [9, 10]. For accurate axial requirements in astigmatism correction, FS-LASIK is favored over SMILE in low to moderate astigmatism correction because the latter requires more alignment of the treatment [11]. Zhang suggested that SMILE should adjust the nomograms for astigmatism correction [12]. In addition, FS-LASIK implementation is more extensive than SMILE. Consequently, the choice for moderate to high astigmatism treatment is generally between FS-LASIK and Toric ICL, with the cornea treated via FS-LASIK and the inside of the eye treated via Toric ICL.

Vector analysis is considered a standard method for analyzing the correction effect of astigmatism by the American National Standards Institute (ANSI) [13]. This method is widely used to evaluate the treatment effect of astigmatism in corneal refractive surgery, cataract phacoemulsification combined with Toric intraocular lens implantation, and Toric ICL implantation, and it can comprehensively assess the effectiveness of astigmatism correction using the treatment magnitude, treatment angle deviation, and so on.

Because the principles of FS-LASIK and Toric ICL are completely different, few studies have compared the effect of astigmatism correction between these surgeries. Therefore, the effect of FS-LASIK and Toric ICL for moderate and high astigmatism correction was compared by vector analysis in our study.

## 2. Materials and Methods

This comparative, randomized, and retrospective study included 44 eyes from 44 subjects who had preoperative astigmatism ranging from −1.00 diopters (D) to −4.50 D [7]. In total, 22 eyes of 22 patients underwent bilateral FS-LASIK, and 22 eyes of 22 patients received bilateral Toric ICL. This prospective study obtained Institutional Review Board approval from the Ethics Committee of the Research Institute of Field Surgery, Daping Hospital of the Army Medical University, Chongqing, China, and it was performed in accordance with the tenets of the Declaration of Helsinki. All of the study participants provided written informed consent.

The common inclusion criteria were as follows: minimum age of 18 years, stable refraction for at least 1 year, myopia with a minimum astigmatism of −1.0 D, corrected distant visual acuity (CDVA) of 20/30 or better, healthy tear film and ocular surface, absence of corneal ectatic diseases, and corneal scars and retinal pathology. Patients using soft and rigid contact lenses were instructed to discontinue use for at least 2 and 4 weeks, respectively. For the Toric ICL patients, the anterior chamber depth was greater than 2.8 mm, and the endothelial cell density was greater than 2000 cells/mm [2]. In addition, for the FS-LASIK patients, the central corneal thickness was more than 500 *μ*m, and the residual stroma was thicker than 280 *μ*m.

### 2.1. Surgical Procedure

For FS-LASIK, the flap was created with a WaveLight FS200 femtosecond laser system (Alcon, USA). The parameters were performed with an intended flap diameter of 8.5 mm, a thickness of 110 *μ*m, a superior hinge, and a side angle cut of 90°. Excimer laser ablation was performed using a WaveLight EX500 Excimer Laser system (Alcon, USA) with an optical zone of 6 mm and pupil tracking technology. The kappa angle of the treatment centration was adjusted based on the Pentacam HR (Oculus Optikgeräte GmbH) before surgery, and the center was fixated intraoperatively.

For Toric ICL implantation, all of the patients underwent 2 preoperative peripheral iridotomies with a neodymium-YAG laser. Patients were marked on the 0°–180°axis using a slit lamp before surgery. After topical anesthesia was administered, a Toric ICL was inserted through a 2.8 mm clear corneal incision with the use of an injector cartridge (STAAR Surgical) after placement of a viscosurgical device (Opegan^TM^; Santen, Osaka, Japan) into the anterior chamber. Then, the Toric ICL was placed in the posterior chamber and rotated to the intended axis using the manipulator.

### 2.2. Data Collection

The examinations included slit-lamp biomicroscopy, CDVA measurement, and subjective refraction before and 3 months after surgery. For all patients, sphere and cylinder were assessed by the same optometrists, and spherical equivalent (SE) values were calculated as the sphere power plus 1/2 of the cylinder power. The eye with the greater cylinder was collected for the study [14]. When both the eyes had an equal cylinder, the right eye was collected.

### 2.3. Vector Analysis

*Preparation for Vector Analysis*. Astigmatism data from the spectacle to the corneal plane were converted, the cylinder axes of the left eyes were flipped around to the vertical axis, and all axis angles were then doubled. The parameters of the vector analysis were as follows: the intended refractive correction (IRC) vector, which is defined as the vector difference between the preoperative astigmatic correction vector and the target postoperative cylinder vector (preoperative − target); the surgically induced refractive correction (SIRC), which is the vector difference between the preoperative and postoperative astigmatic correction vectors (preoperative − postoperative); the error vector (EV), which is defined as the vector difference between the intended refractive correction and the surgically induced refractive correction (IRC-SIRC); the error ratio (ER), which is the proportion of the intended correction that was not successfully treated (|EV|/|IRC|); the correction ratio (CR), which is the ratio of the achieved correction magnitude to the required correction (|SIRC|/|IRC|); the error of magnitude (EM), which is the arithmetic difference of the magnitudes between SIRC and IRC (|IRC|-|SIRC|); and the error of angle (EA), which measures whether the treatment was applied at the correct axis.

### 2.4. Statistical Analysis

The statistical analysis was performed using PASW software V.18.0 (SPSS/IBM, Chicago, Illinois, USA). The Kolmogorov–Smirnov test was used to analyze the normality of the parameters. Independent sample *T*-test was used to normal distribution variables, and Mann–Whitney *U* test was used to abnormal distribution variables between groups. Categorical variables were evaluated using the *χ*^2^ test. A value of *p* < 0.05 was considered statistically significant.

## 3. Results

Overall, 44 eyes (44 patients) were included in the study, with 22 eyes in the FS-LASIK group and 22 eyes in the Toric ICL group. The mean age of the patients was 22.72 ± 4.85 years and 25.27 ± 5.49 years in FS-LASIK and Toric ICL groups, respectively. Significant differences were not observed in the manifest sphere, manifest cylinder, manifest spherical equivalent, and axial and CDVA between two groups preoperatively or postoperatively (Table 1). All surgeries were uneventful, with no intraoperative complications.

### 3.1. Safety and Effectiveness Analysis

At 3 months after surgery, 6 (27.27%) eyes in the FS-LASIK group and 6 (27.27%) eyes in the Toric ICL group gained ≥1 line in the CDVA. In both groups, none of the eyes lost ≥2 lines in the CDVA.

**Figure 1:**
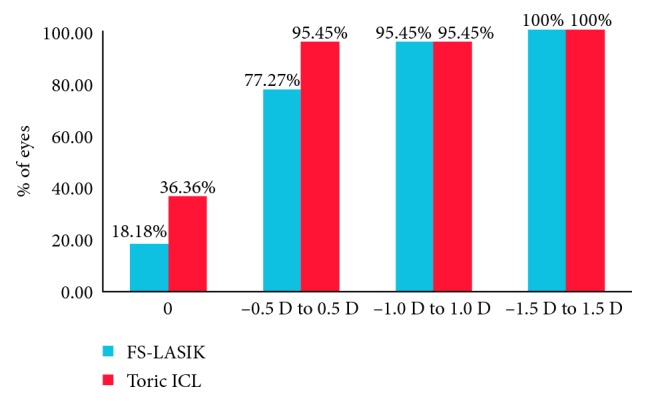
Postoperative spherical equivalent in dioptres (D) between FS-LASIK and Toric ICL.

**Figure 2:**
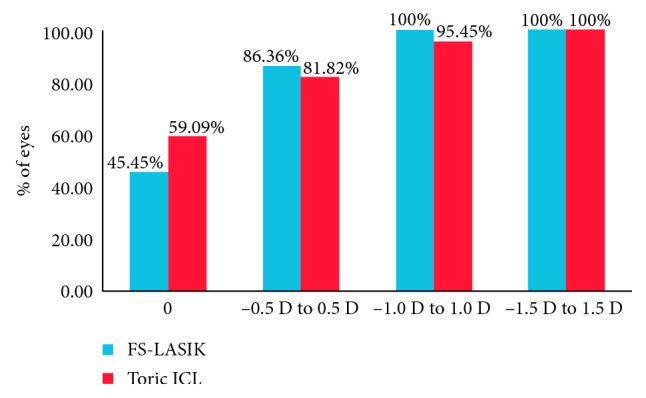
Postoperative refractive astigmatism in dioptres (D) between FS-LASIK and Toric ICL.

The percentage of the eyes with postoperative SE within 0 D, ±0.5 D, ±1.0 D, and ±1.5 D was 18.18% and 36.36%, 77.27% and 95.45%, 95.45% and 95.45%, and 100% and 100% in the FS-LASIK group and Toric ICL group, respectively (Figure 1). The percentage of the eyes with postoperative refractive astigmatism within 0 D, ±0.5 D, ±1.0 D, and ±1.5 D was 45.49% and 59.09%, 86.36% and 81.82%, 100% and 95.45%, and 100% and 100% in the FS-LASIK group and Toric ICL group, respectively (Figure 2). Both the postoperative SE and postoperative refractive astigmatism were not significantly different between the two groups (*χ*^2 ^= 4.27 and 1.09; *p*=0.12 and 0.58, respectively).

### 3.2. Vector Analysis

The vector analysis results showed that significant differences did not occur in the IRC, SIRC, EV, CR, ER, EM, and EA between the FS-LASIK and Toric ICL groups (Table 2). Significant differences in these parameters were not observed between the two groups.

**Figure 3:**
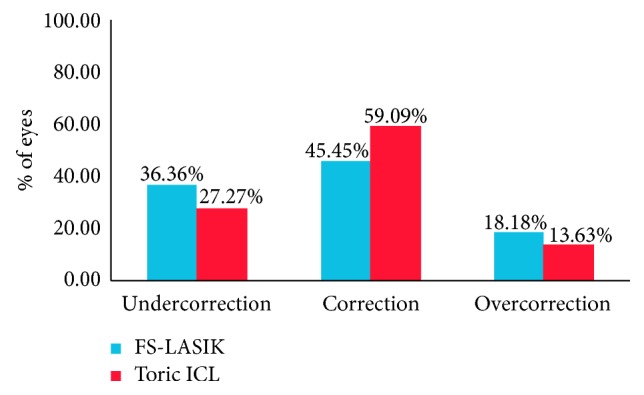
Astigmatism correction ratio in FS-LASIK and Toric ICL.

The percentage of the undercorrection and overcorrection was 36.36% and 27.27% and 18.18% and 13.63% between the FS-LASIK and Toric ICL groups, respectively (Figure 3), and significant differences were not observed (*χ*^2^ = 0.82, *p*=0.66).

**Figure 4:**
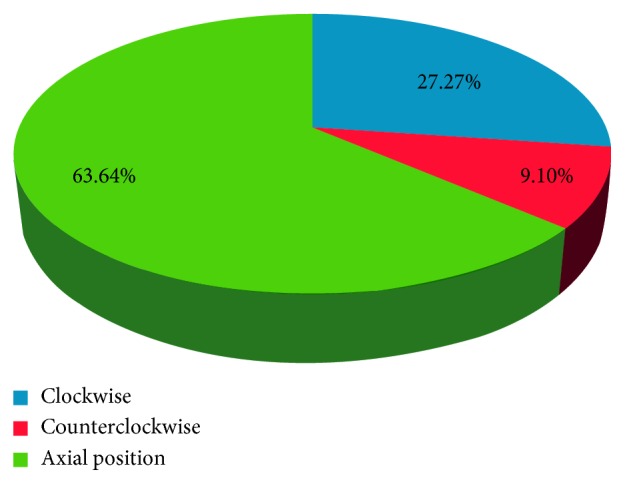
Correct axis between the achieved treatment and the intended treatment in FS-LASIK.

**Figure 5:**
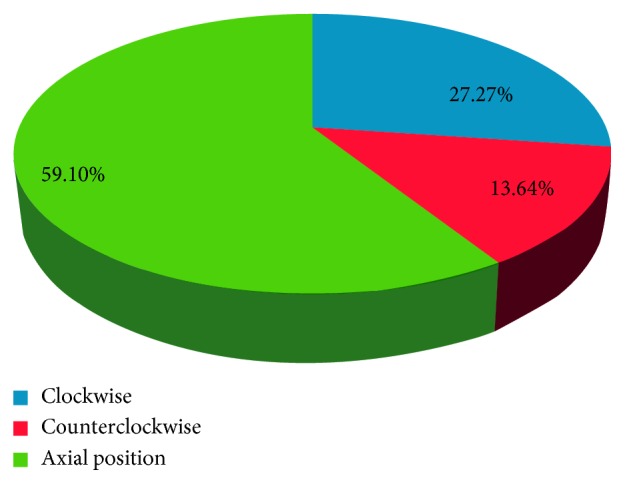
Correct axis between the achieved treatment and the intended treatment in Toric ICL.

The percentage of correct axes between the achieved treatment and the intended treatment (clockwise and counterclockwise) was 27.27% and 27.27% and 9.1% and 13.64% between the FS-LASIK and Toric ICL groups, respectively (Figures 4 and 5), and significant differences were not observed (*χ*^2^ = 0.24, *p*=0.88). However, the error angles were 4.11 ± 3.02 (1–10) and 8.11 ± 3.82 (2–14) in the off-axis correction of the LASIK and Toric ICL groups. A significant difference was observed between the groups (*t *= −2.46, *p*=0.02).

## 4. Discussion

In this study, our findings suggest that FS-LASIK and Toric ICL presented good safety and efficacy in the treatment of moderate and high astigmatism, which is consistent with the results of previous studies. The CDVA of the patients was improved in both groups, and none of the patients lost CDVA. High astigmatism may induce high-order aberrations [15], and high astigmatism corrected using spectacles may generate imaging distortion, which can reduce the patient's CDVA. Topography-guided FS-LASIK and Toric ICL can reduce high-order aberrations [16, 17]. Similarly, correcting myopia and astigmatism also reduces the imaging distortion from spectacles. However, FS-LASIK and Toric ICL have different treatment characteristics. In FS-LASIK, corneal ablation occurs via excimer laser spot superposition at high speeds to achieve therapeutic purposes. In the excimer laser ablation procedure, changes in the circumambient temperature and humidity, attenuation of laser energy, and movement of the laser cutting plane can affect the treatment effect. Toric ICL is a molded lens designed for therapeutic purposes, and it is implanted in the posterior chamber; thus, deviations from the pupil center are rare. The therapeutic gradient of Toric ICL for astigmatism is 0.5D, while FS-LASIK is usually 0.25 D. In addition, undercorrection is more likely to occur with Toric ICL. For high astigmatism, the treatment may have to be adjusted according to nomograms. Different devices usually have different nomograms, and the success of the adjustment depends on the experience of the surgeon. Although different advantages and limitations were observed between the two surgeries, significant differences were not observed in the correction effect. Ganesh et al. found that the predictability of low to moderate astigmatism correction was not significantly different among FS-LASIK, Toric ICL, and reflex SMILE [17]. Hasegawa et al. found that the predictability in the LASIK group was higher in the moderate refractive cylinder but lower in the high refractive cylinder than that in the Toric phakic intraocular lens group [18]. Therefore, we believe that there are similar treatment effects in moderate to high astigmatism between FS-LASIK and Toric ICL.

Astigmatism correction is based on the magnitude of the astigmatism and on the axis of the astigmatism, which makes the treatment of astigmatism more complicated compared with the treatment of myopia. Alpins found that when the treatment is off the intended axis, then the effect of the astigmatism correction will be reduced [19]. As a result, accurate axial correction is a difficulty associated with astigmatism correction. Zhang et al. found that axial errors may be one of the potential factors for moderate and high astigmatism undercorrection [20]. Our study also found that the patients showing undercorrection or overcorrection had different degrees of axial errors in the treatment according to the vector analysis, and axial errors were not observed in the complete correction cases. Pupil tracking avoided decentration ablation, which resulted from eye movement in FS-LASIK, although the patient's head position and rotation or vertical movement of the eye may still cause off-axis ablation. The treatment must be adjusted according to the nomogram for high astigmatism correction via FS-LASIK, and overcorrection may be related to fewer adjustments for corrections. The effective lens position and posterior corneal astigmatism might represent influencing factors in Toric ICL when the Toric intraocular lens is used to correct for astigmatism in cataract surgery [21].

To our knowledge, few studies have compared the axial offset degree between FS-LASIK and Toric ICL. In our study, we found that the angle error of Toric ICL was greater than that of FS-LASIK in patients with axial migration because axial errors may occur in the FS-LASIK procedure, although the influence of such errors on astigmatism correction may be reduced with the strict supervision of the surgeon. However, the Toric ICL axis was marked before surgery and found to be consistent with the expected intraoperative axis by the surgeons. Nonetheless, Toric ICL rotation is an uncertain postoperative scenario, and repositioning is required when severe rotation occurs. Therefore, we believe that the error angle of Toric ICL may be greater than that of FS-LASIK in patients with off-axis correction.

## 5. Conclusions

In conclusion, our study found that FS-LASIK and Toric ICL were effective for moderate and high astigmatism correction. However, Toric ICL might create a larger error angle than FS-LASIK when off-axis correction occurs.

## Figures and Tables

**Table 1:** Demographic data of the FS-LASIK and Toric ICL groups.

	FS-LASIK group	Toric ICL group	*p* value
Patients/eyes (*n*)	22/22	22/22	—
Eye, right (%)	54.55	40.91	0.36^*∗∗*^
Sex, male (%)	50	31.82	0.38^*∗∗*^
Age (*y*)	22.72 ± 4.85 (18 to 33)	25.27 ± 5.49 (18 to 35)	0.11^*∗*^
Presphere (D)	−6.97 ± 1.20 (−9.25 to −5.00)	−8.32 ± 3.14 (−14.00 to −2.00)	0.07^*∗*^
Precylinder (D)	−2.40 ± 0.59 (−3.50 to −1.25)	−2.28 ± 0.88 (−4.00 to −1.00)	0.57^*∗*^
Pre-SE (D)	−8.18 ± 1.10 (−10.25 to −6.75)	−9.47 ± 2.99 (−15.00 to −3.75)	0.07^*∗*^
Preaxial	17.50/5.00/172.50 (0 to 175)	5.00/0.00/78.75 (0 to 175)	0.06^#^
Pre-CDVA (logMAR)	0.04 ± 0.06 (0 to 0.2)	0.06 ± 0.09 (0 to 0.3)	0.31^*∗*^
Postsphere (D)	−0.08 ± 0.40 (−1.00 to 0.75)	0.13 ± 0.49 (−0.5 to 2.00)	0.14^*∗*^
Postcylinder (D)	−0.19 ± 0.33 (−1.00 to 0.25)	−0.27 ± 0.37 (−1.25 to 0)	0.45^*∗*^
Post-SE (D)	−0.18 ± 0.45 (−1.25 to 0.75)	−0.01 ± 0.43 (−0.5 to 1.38)	0.23^*∗*^
Postaxial	0.00/0.00/85.00 (0 to 155)	0.00/0.00/46.25 (0 to 170)	0.36^*∗*^
Post-CDVA (logMAR)	0.01 ± 0.03 (0 to 0.1)	0.02 ± 0.04 (0 to 0.1)	0.43^#^

Pre: preoperative values; SE: spherical equivalent; normal distribution variables: mean ± SD (range); abnormal distribution variables: median/Q25/Q75 (range); ^*∗∗*^chi-squared test; ^*∗*^independent sample *T*-test; ^*#*^Mann–Whitney *U* test.

**Table 2:** Vector parameters for FS-LASIK and Toric ICL.

	FS-LASIK group (*n*=22)	Toric ICL group (*n*=22)	*p* value
IRC	2.00 ± 0.50 (1.01 to 3.00)	1.86 ± 0.76 (0.77 to 1.86)	0.47^*∗*^
SIRC	1.95 ± 0.48 (1.01 to 3.00)	1.76 ± 0.81 (0.77 to 3.30)	0.36^*∗*^
EV	0.25/0.00/0.31 (0 to 0.97)	0.00/0.00/0.49 (0 to 1.29)	0.86^#^
CR	1.00/0.91/1.00 (0.69 to 1.28)	1.00/1.00/0.92 (0.41 to 1.21)	0.96^#^
ER	0.12/0.00/0.19 (0 to 0.39)	0.00/0.00/0.30 (0 to 0.61)	0.92^#^
EM	0.00/0.00/0.17 (−0.47 to 0.77)	0.00/0.00/0.22 (−0.35 to 1.24)	0.98^#^
EA	0.00/−2.00/0.00 (−10 to 8)	0.00/−4.25/0.00 (−14 to 13)	0.81^#^

RC: intended refractive correction; SIRC: surgically induced refractive correction; EV: error vector; CR: correction ratio; ER: error ratio; EM: error of the magnitude; normal distribution variables: mean ± SD (range); abnormal distribution variables: median/Q25/Q75 (range); ^#^Mann–Whitney *U* test; ^*∗*^independent sample *T*-test.

## Data Availability

The data used to support the findings of this study are included within the article.
